# Short-Term Clinical Efficacy of Neoadjuvant Chemotherapy Combined With Laparoscopic Gastrectomy for Locally Advanced Siewert Type II and III Adenocarcinoma of the Esophagogastric Junction: A Retrospective, Propensity Score-Matched Study

**DOI:** 10.3389/fonc.2021.690662

**Published:** 2021-09-29

**Authors:** Qing Feng, Du Long, Ming-shan Du, Xiao-song Wang, Zhen-shun Li, Yong-liang Zhao, Feng Qian, Yan Wen, Pei-wu Yu, Yan Shi

**Affiliations:** ^1^ Department of General Surgery, The First Affiliated Hospital, Army Medical University, Chongqing, China; ^2^ Radiology Department, The First Affiliated Hospital, Army Medical University, Chongqing, China

**Keywords:** esophagogastric junction, neoadjuvant chemotherapy, laparoscopic, postoperative complication, Siewert II and III

## Abstract

**Background:**

Laparoscopic gastrectomy (LG) has been increasingly used for the treatment of locally advanced Siewert type II and III adenocarcinoma of the esophagogastric junction (AEG). However, whether LG can achieve the same short-term efficacy in the treatment of patients who receive neoadjuvant chemotherapy (NACT) remains controversial. Thus, the aim of this study was to investigate the clinical outcomes of NACT combined with LG for Siewert type II and III AEG.

**Methods:**

This retrospective study identified patients with locally advanced Siewert type II and III AEG diagnosed between May 2011 and October 2020 using the clinical tumor-node-metastasis (cTNM) staging system. The short-term outcomes were compared between the matched groups using a 1:3 propensity score matching (PSM) method, which was performed to reduce bias in patient selection.

**Results:**

After PSM, 164 patients were selected, including 41 in the NACT group and 123 in the LG group. The baseline characteristics were similar between the two groups. Compared with the LG group, the NACT group exhibit a smaller tumor size and significantly less advanced pathological tumor classification and nodal classification stages. The time to first flatus of the NACT group was significantly shorter, but the hospital stay was significantly longer than that of the LG group. The NACT group showed similar overall (29.3% *vs* 25.2%, P=0.683), systemic (24.4% *vs* 21.1%, P=0.663), local (12.2% *vs* 9.8%, P=0.767), minor (19.5% *vs* 19.5%, P=1.000) and major (9.8% *vs* 5.7%, P=0.470) complications as the LG group. Subgroup analyses showed no significant differences in most stratified parameters. Operation time≥ 300 minutes was identified as an independent risk factor for overall complications. Age≥ 60 years was identified as an independent risk factor for major complications.

**Conclusion:**

NACT combined with LG for AEG does not increase the risk of postoperative morbidity and mortality compared with LG.

## Introduction

The incidence of adenocarcinoma of the esophagogastric junction (AEG) is rapidly increasing, especially Siewert II and III AEG (1, 2). Surgery remains the only radical cure for AEG (3). Since laparoscopic gastrectomy (LG) was first introduced by Kitano in 1994 (4), it has been widely used for early gastric cancer and advanced gastric cancer with the advantages of less injury, faster recovery, and lower morbidity of postoperative complications (5–8). For Siewert type II and III AEG, Liao’s meta-analysis (9) revealed that LG can achieve short-term surgical outcomes comparable to open gastrectomy (OG). However, the development of surgical procedures did not improve long-term outcomes (10). In addition, due to the special location of this tumor, most cases are diagnosed at an advanced stage (11), seriously impacting on the prognosis of patients and resulting in a lower overall survival.

Accumulating evidence has revealed that neoadjuvant therapy improves the efficacy of AEG compared with surgery alone (12–14). However, chemotherapy-induced tissue fibrosis and oedema provide new technical challenges for minimally invasive procedures and increase the difficulty of the operation. It remains controversial whether LG is suitable for AEG patients after NACT. Therefore, we conducted a single-centre retrospective, propensity score-matched study to determine whether LG is suitable for AEG patients after NACT.

## Materials and Methods

### Patients

A total of 256 Siewert type II or III AEG patients who underwent laparoscopic gastrectomy were identified from a prospectively maintained database containing all gastric cancers diagnosed at The First Affiliated Hospital of Army Medical University in China between May 2011 and October 2020. The decision for NACT was discussed in the Department of General Surgery and determined by the patients who were informed of the possible complications of the procedure and the potential benefits and harms of NACT compared with the LG approach. Written informed consent was obtained from all patients before the operation.

The inclusion criteria were as follows: patients aged 18 to 85 years who were diagnosed with Siewert type II/III AEG by computed tomography (CT); patients who received gastroscopy and were pathologically confirmed by postoperative biopsy; patients who adopted a complete trans-abdominal approach; patients with no distant metastasis or invasion to adjacent organs; and patients who underwent D2 radical laparoscopic gastrectomy. The exclusion criteria included non-radical operation, emergent operation previous gastrectomy, endoscopic mucosal resection, or endoscopic submucosal dissection. In total, 41 and 192 patients were included in the NACT and LG groups, respectively. Clinical stage was evaluated for all patients using intravenous contrast-enhanced CT before and after NACT. Before the study was conducted, CT data were evaluated by a professional radiologist who was blinded to the clinical information of the patient. This study was approved by the Ethics Committee of the First Affiliated Hospital of Army Medical University, PLA (Approval number: KY2021059).

### Neoadjuvant Chemotherapy and Evaluation of Clinical Response and Toxicity

Patients received different cycles of NACT preoperatively, and a median of 3 (2, 4) cycles was administered. Among the 41 patients in the NACT group, 37 (90.2%) received the SOX (oxaliplatin + S-1) regimen, 2 (4.9%) received the XELOX (oxaliplatin + capecitabine) regimen, and 2 (4.9%) received the FOLFOX (oxalipatin + fluorouracil + leucovorin) regimen. The toxicity and adverse events of NACT were evaluated according to the World Health Organization (WHO) standard criteria (15). The response to chemotherapy was endoscopically and radiologically evaluated by endoscopy and CT scans. Post-NACT evaluation of the target lesions was divided into four categories: complete remission (CR), partial remission (PR), stable disease (SD), and progressive disease (PD) according to the Response Evaluation Criteria in Solid Tumours (RECIST, version 1.1) (16).

### Surgery and Postoperative Outcome

Patients in the NACT group underwent radical gastrectomy after the completion of NACT (3-4 weeks). All patients who underwent laparoscopic gastrectomy with D2 lymphadenectomy were treated by three experienced surgeons according to the Japanese Gastric Cancer Treatment Guidelines (17, 18). Specific surgical gastrectomy procedures, including proximal and total gastrectomy, were selected depending on the location of the primary tumor. Reconstruction of the gastrointestinal tract was performed according to the type of gastrectomy. Postoperative outcomes, including the results of the pathological outcomes, postoperative recovery (i.e., the times to first flatus and length of overall and postoperative hospital stay), and morbidity and mortality rates, were evaluated. Pathologic evaluations and staging were updated according to the 8th American Joint Committee on Cancer (AJCC) TNM staging system (19). Postoperative complications were defined as complications that occurred within 30 days after surgery. One month after the operation, outpatient and telephone follow-ups were conducted to determine the survival and severity of the patients after discharge.

### Statistical Analysis

To minimize the bias between the NACT group and the LG group, we performed PSM with the R (x64 3.5.0) MatchIt package. Age, sex, body mass index (BMI) on admission, American Society of Anaesthesiologists (ASA) grade, Siewert classification, cT stage, cN stage, cTNM stage, resection range and tumor differentiation were chosen to perform 1:3 matching using the “nearest” method. Data are presented as proportions for categorical variables and as the mean ± SD for continuous variables. Variables with high skew are presented as the median (IQR). Categorical variables were compared using the χ2 test or Fisher’s exact test, whereas continuous variables were compared using Student’s t-test or the Mann-Whitney U test. Variables with P-values<0.10 in univariate analysis were included in the multivariate analysis. Multivariate analysis was conducted with the binary logistic regression model to identify independent risk factors for postoperative complications. A P-value (two-sided)<0.05 was considered statistically significant. Data analyses were conducted using SPSS (IBM Corp. Released 2017. IBM SPSS Statistics for Windows, Version 26.0. Armonk, NY: IBM Corp).

## Results

### Patients’ Characteristics

**Figure 1:**
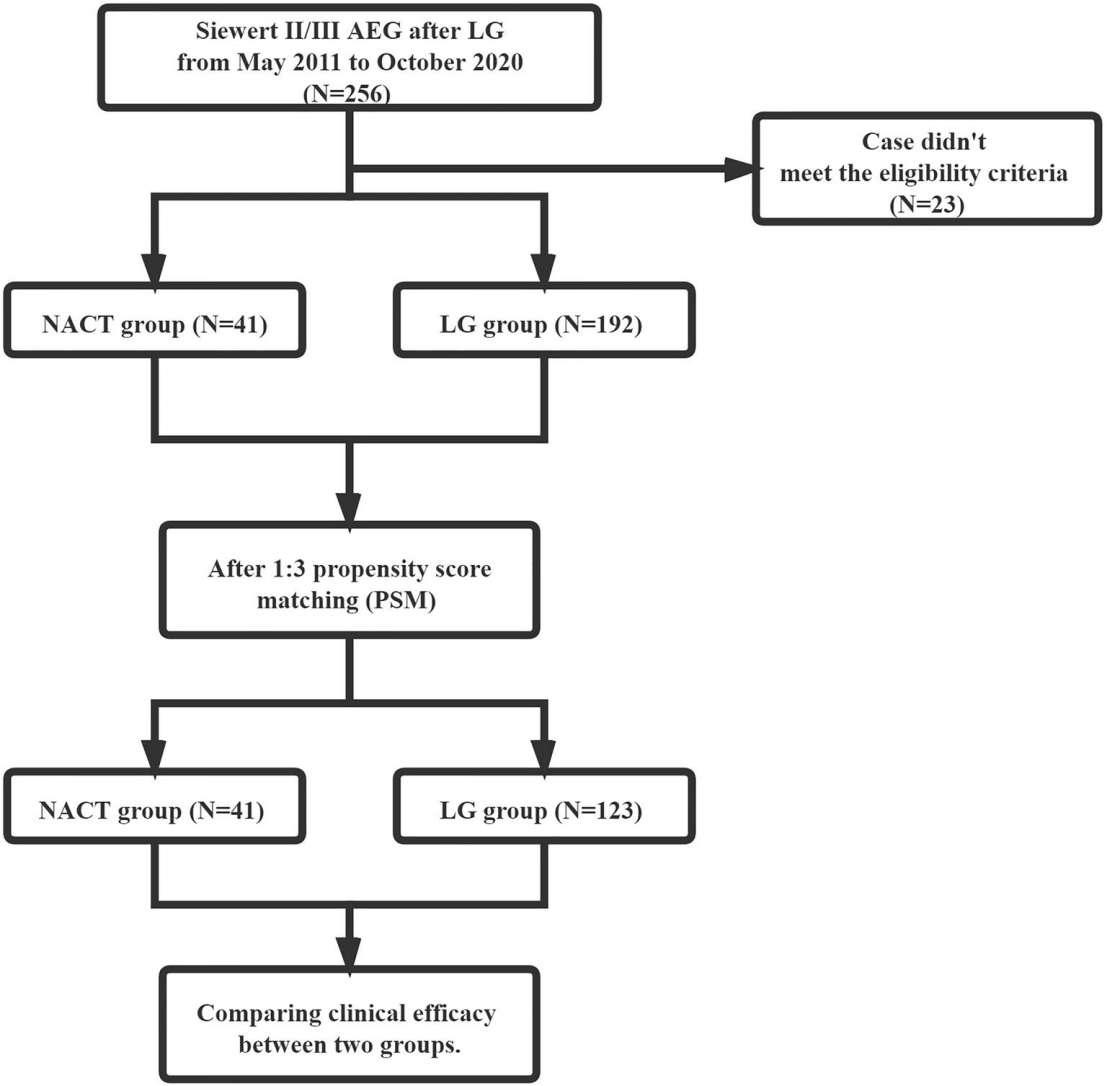
Patient selection diagram based on PSM.

Flow of patient enrolment is presented in **Figure 1**. **Table 1** summarizes the clinicopathological characteristics of the patients in the two groups. Clinical T stage, clinical N stage and tumor differentiation significantly differed between the NACT and LG groups. On the basis of 1:3 PSM, 164 patients (41 in the NACT group and 123 in the LG group) were selected for analysis. After PSM, no significant differences in age, sex, BMI on admission, ASA, Siewert classification, cT stage, cN stage, cTNM stage, resection range and tumor differentiation were noted between the two groups.

**Table 1:** Characteristics of patients before and after PSM.

Characteristic	Crude Cohort (n = 233)			PSM Cohort (n = 123)		
	NACT group	LG group	P value	NACT group	LG group	P value
	(n = 41)	(n = 192)		(n = 41)	(n = 123)	
**Age (years)**			0.202			0.991
<60	20	73		20	55	
≥60	21	119		21	68	
**Sex**			0.615			0.663
Male	31	152		31	97	
Female	10	40		10	26	
**BMI (kg/m2) on admission**			0.614			0.695
<18.5	4	11		2	9	
≥18.5 and <25	30	146		30	96	
≥25	7	35		9	18	
**ASA score**			***0.025***			0.207
1	32	111		32	85	
2	8	78		8	37	
3	1	3		1	1	
**Siewert classification**			0.145			0.278
Type II	16	99		16	60	
Type III	25	93		25	63	
**Clinical T stage**			0.078			0.399
T2	0	20		0	1	
T3	13	46		13	25	
T4a	28	126		28	97	
**Clinical N stage**			***0.014***			0.562
0	0	37		0	0	
1	10	46		10	30	
2	22	69		22	56	
3	9	40		9	37	
**Clinical TNM stage**			***0.008***			1.000
I	0	15		0	0	
IIA	0	4		0	0	
IIB	0	22		0	0	
III	41	151		41	123	
**Resection range**			0.056			1.000
Proximal	1	26		1	2	
Total	40	166		40	121	
**Differentiation**			***0.025***			0.349
Well/moderately	12	93		12	48	
Poorly/undifferentiated	29	99		29	75	

PSM, propensity score matching; NACT, neoadjuvant chemotherapy; BMI, body mass index; ASA, American Society of Anesthesiologists. Italicized and bold values represent significant differences.

### NACT Response and Toxicity Analysis

In this study, 27 (65.8%) patients exhibited PR, 12 (29.3%) exhibited SD, and 2 (4.9%) patients exhibited PD according to contrast-enhanced CT before and after NACT (**Table 2**). The BMI of the NACT group after NACT was significantly greater than that on admission (22.50 *vs* 21.90 P=0.016). 8 (19.5%) of the 41 treated patients experienced at least grade 3-4 toxicity during NACT treatment. The most common grade 3-4 toxicities were leukopenia/neutropenia (9.8%) and nausea and vomiting (12.2%) (**Table 2**).

**Table 2:** Characteristics of neoadjuvant chemotherapy, n (%).

Chemotherapy regimen	
SOX	37 (90.2%)
FOLFOX	2 (4.9%)
XELOX	2 (4.9%)
**Cycles of NACT completed**	
2 cycles	10 (24.4%)
3 cycles	19 (46.3%)
4 cycles	7 (17.1%)
More than 4 cycles	5 (12.2%)
**Clinical response per RECIST criteria**	
PR	27 (65.8%)
SD	12 (29.3%)
PD	2 (4.9%)
**Grade 3 or 4 adverse effects**	
Leukopenia/neutropenia	4 (9.8%)
Nausea/vomiting	5 (12.2%)
Skin disease	2 (4.9%)
Anaemia	1 (2.4%)
Thrombocytopenia	1 (2.4%)
**Chemotherapy-surgical procedure interval, week (median, IQR)**	4 (3,6)

NACT, neoadjuvant chemotherapy; PR, partial remission; SD, stable disease; PD, progressive disease. IQR, interquartile range.

### Comparison of Operative Findings

The proximal margin of one patient in the NACT group and four patients in the LG group was found to be positive. R0 resection was performed for 97.8% of patients in the NACT group and 93.5% of patients in the LG group (P = 0.453). The amount of blood loss, transfused patient number, and operation time were comparable between the two groups. During the procedure, 6 patients (14.6%) in the NACT group were converted to open gastrectomy, whereas 12 patients (9.8%) in the LG group showed no significant differences (P=0.395). No statistically significant difference was found between the two groups regarding the length of incision, distal margin or proximal margin (**Table 3**). After PSM, the median time to first flatus of the NACT group was significantly shorter than that of the LG group (3 *vs* 4 days, P=0.004). Both the total hospital stay and postoperative hospital stay of the NACT group were significantly longer than those of the LG group.

**Table 3:** Comparison of operative and postoperative parameters between the NACT group and LG group, n (%).

Variable	NACT group	LG group	P value
	(n = 41)	(n = 123)	
**Resection**			0.453
R0	40 (97.8%)	115 (93.5%)	
R1	1 (2.4%)	8 (6.5%)	
**Operation time, min (mean ± SD)**	280.34 ± 53.61	273.73 ± 48.87	0.466
**Blood loss, ml (median, IQR)**	150 (100, 200)	160 (110, 200)	0.480
**Blood transfusion**			1.000
Yes	5 (12.2%)	13 (10.6%)	
No	36 (87.8%)	108 (89.4%)	
**Lymph node dissection range**			0.640
D2	39 (95.1%)	119 (96.7%)	
D2+	2 (4.9%)	4 (3.3%)	
**Conversion to open from laparoscopic gastrectomy**	6 (14.6%)	12 (9.8%)	0.395
**Length of incision, cm (median, IQR)**	7 (6,9)	6 (5,8)	0.070
**Distal margin, cm (median, IQR)**	8 (5,14.5)	8 (5,10)	0.306
**Proximal margin, cm (median, IQR)**	3 (2,4.5)	3 (2,3)	0.161
**Tumor size, cm (median, IQR)**	3 (2,4.5)	4 (3,5)	***<0.001***
**The number of resected lymph nodes (mean ± SD)**	30.54 ± 14.20	33.68 ± 13.42	0.202
**The number of metastatic lymph nodes (median, IQR)**	0 (0,4)	5 (1,9)	***<0.001***
**Pathological tumor classification**			***<0.001***
(y)pT0-2	16 (39.0%)	14 (11.4%)	
(y)pT3	0	2 (1.6%)	
(y)pT4a/4b	25 (61.0%)	107 (87.0%)	
**Pathologic nodal classification**			***<0.001***
(y)pN0	22 (53.7%)	13 (10.6%)	
(y)pN1	7 (17.1%)	31 (25.2%)	
(y)pN2	8 (19.5%)	28 (22.8%)	
(y)pN3	4 (9.8%)	37 (30.1%)	
**Total hospital stay, d (median, IQR)**	18 (14,22)	15 (13,18)	***0.012***
**Postoperative hospital stay, d (median, IQR)**	11 (9,14)	9 (8,11)	***0.003***
**Time to first flatus, d (median, IQR)**	3 (3,4)	4 (3,5)	***0.004***

NACT, neoadjuvant chemotherapy; LG, laparoscopic gastrectomy; SD, standard deviation; IQR, interquartile range. Italicized and bold values represent significant differences.

### Analyses of Pathological Outcomes

The average number of harvested lymph nodes (LNs) did not significantly differ (P=0.225) in the NACT (30.54) and LG groups (33.51), whereas the number of metastatic LNs was significantly lower in the NACT group (**Table 3**). The tumor size of the NACT group was smaller than that of the LG group (P<0.001). Following PSM, both the (y)pT and (y)pN stage categories of the NACT group were significantly less advanced than those of the LG group.

### Analyses of Postoperative Complications

The postoperative morbidity and mortality of patients in the PSM cohort are shown in **Table 4**. Morbidity was comparable between the two groups (29.3% *vs* 25.2%, P=0.683). No differences in systemic complications (24.4% *vs* 21.1%, P=0.663) and local complications (12.2% *vs* 9.8%, P=0.767) were noted between the groups. No significant differences in the comparison of specific complications (all P>0.05) were noted between the groups. More infectious complications were noted in the NACT group compared with the LG group; however, the difference was not significant (24.4% *vs* 18.7%, P=0.431). No significant differences in complication severity according to the Clavien-Dindo grade were noted (20, 21). Four patients (9.8%) in the NACT group and 7 patients (5.7%) in the LG group experienced grade III or higher complications (P=0.470). One patient in the NACT group and 2 patients in the LG group underwent reoperation due to abdominal bleeding.

**Table 4:** Postoperative Complications of the patients in NACT group and LG group, n (%).

Variable	NACT group	LG group	P-value
	(n = 41)	(n = 123)	
**Postoperative complications**	12 (29.3%)	31 (25.2%)	0.683
**Systemic complication**	10 (24.4%)	26 (21.1%)	0.663
Heart failure	1 (2.4%)	1 (0.8%)	0.439
Respiratory failure	2 (4.9%)	4 (3.3%)	0.640
Pulmonary infection	6 (14.6%)	17 (13.8%)	0.897
Pleural effusion	3 (7.3%)	11 (8.9%)	1.000
Urinary tract infection	0	1 (0.8%)	1.000
Hepatic malfunction	0	3 (2.4%)	1.000
**Local complication**	5 (12.2%)	12 (9.8%)	0.767
Duodenal stump leakage	1 (2.4%)	1 (0.8%)	0.439
Anastomotic leakage	2 (4.9%)	2 (1.6%)	0.260
Intra-abdominal infection	3 (7.3%)	5 (4.1%)	0.414
Abdominal bleeding	0	4 (3.3%)	0.573
Wound infection	0	2 (1.6%)	1.000
**Infectious complication**	10 (24.4%)	23 (18.7%)	0.431
**Clavien-Dindo Classification**			
Grades I-II	8 (19.5%)	24 (19.5%)	1.000
Grade I	1 (2.4%)	4 (3.3%)	1.000
Grade II	7 (17.1%)	20 (16.3%)	0.707
Grades III-V	4 (9.8%)	7 (5.7%)	0.470
Grade III	1 (2.4%)	3 (2.4%)	1.000
Grade IV	3 (7.3%)	4 (3.3%)	0.368
Grade V	0	0	NA
**Reoperation**	1 (2.4%)	2 (1.6%)	1.000

NACT, neoadjuvant chemotherapy; LG, laparoscopic gastrectomy; NA, not available.

### Subgroup Analyses

**Figure 2:**
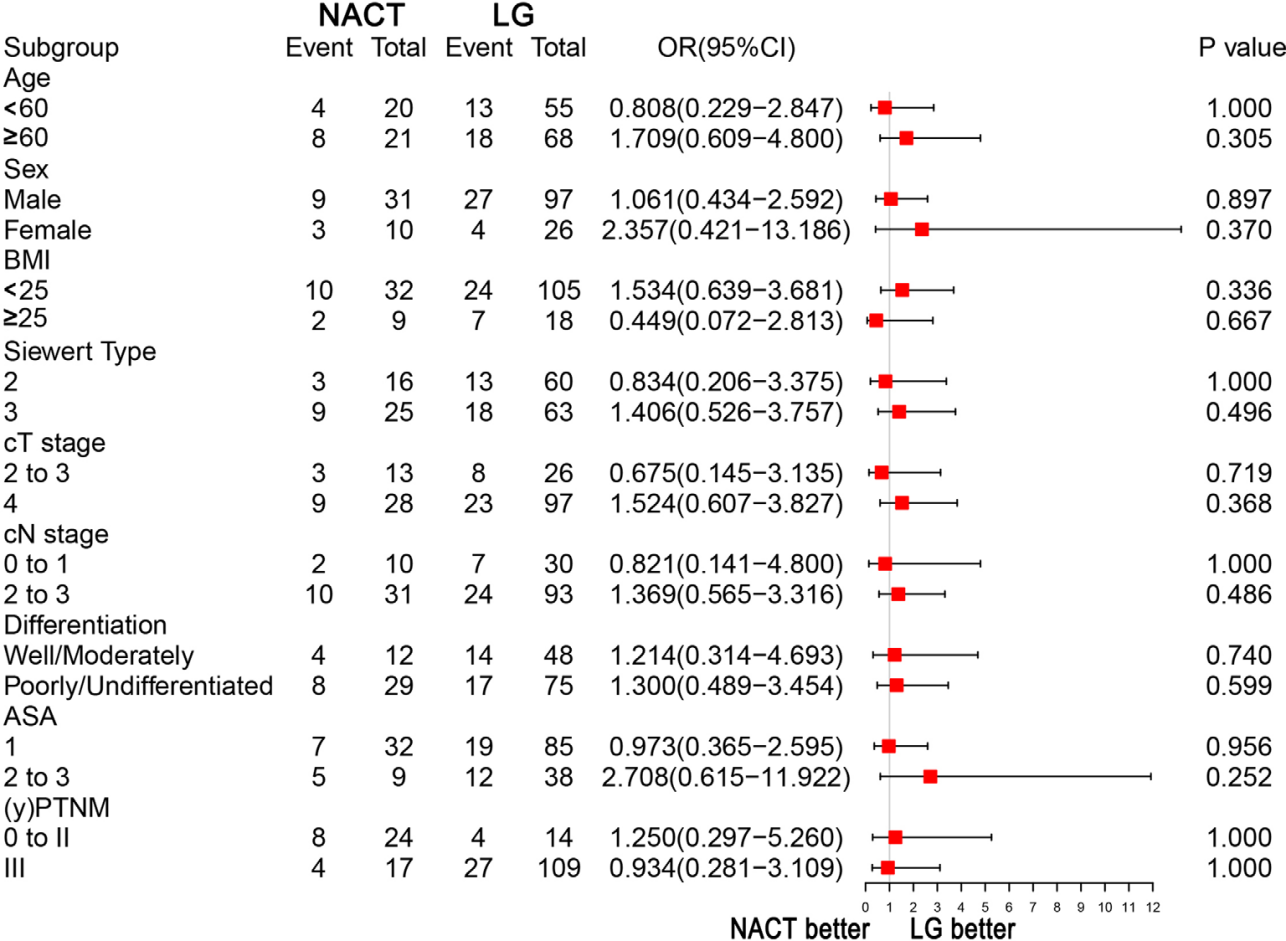
Subgroup analyses of overall complications.

Subgroup analyses were performed for overall complications in the PSM cohort. No significant differences in any stratified parameters in terms of overall complications were noted between the two groups (**Figure 2**).

### Risk Factor for Overall and Major Complications

Univariate analysis showed that BMI ≥25, BMI <18.5, operation time≥ 300 minutes and blood loss ≥200 ml were positively correlated with overall complications (**Table 5**). Multivariate analysis revealed that operation time≥ 300 minutes (P=0.049) was an independent risk factor for overall complications (**Table 5**). Regarding major complications, age≥ 60 years, operation time≥ 300 and blood loss ≥200 were correlated with major complications in univariate analysis. In multivariate analysis, age≥ 60 years (P=0.042) was identified as an independent risk factor for major complications.

**Table 5:** Analysis of risk factors for overall and severe complications in the crude cohort.

Variables	Overall complication				Severe complication			
	Univariate		Multivariate		Univariate		Multivariate	
	OR (95% CI)	P value	OR (95% CI)	P value	OR (95% CI)	P value	OR (95% CI)	P value
**Sex**								
Male	Ref				Ref			
Female	0.711 (0.338-1.493)	0.367			0.771 (0.212-2.794)	0.692		
**Age (years)**								
<60	Ref				Ref		Ref	
≥60	1.110 (0.613-2.010)	0.730			3.333 (0.931-11.940)	***0.064***	3.881 (1.051-14.330)	***0.042***
**ASA Grade**								
I	Ref				Ref			
II-III	1.605 (0.892-2.888)	0.115			1.511 (0.561-4.073)	0.414		
**BMI**								
≥18.5 and <25	Ref		Ref		Ref			
<18.5	2.864 (0.912-8.999)	***0.072***	3.011 (0.925-9.801)	0.067	3.055 (0.595-15.686)	0.181		
≥25	2.056 (1.007-4.199)	***0.048***	1.886 (0.910-3.910)	0.088	2.270 (0.733-7.035)	0.155		
**Neoadjuvant chemotherapy**								
No	Ref				Ref			
Yes	1.144 (0.543-2.410)	0.723			1.489 (0.460-4.821)	0.507		
**Siewert type**								
II	Ref				Ref			
III	1.310 (0.733-2.342)	0.362			1.429 (0.524-3.891)	0.485		
**Clinical TNM stage**								
I-II	Ref				Ref			
III	0.661 (0.321-1.361)	0.261			0.672 (0.207-2.176)	0.507		
**Histopathological grade**								
Well/moderately	Ref				Ref			
Poorly/undifferentiated	0.868 (0.486-1.551)	0.633			1.551 (0.554-4.346)	0.403		
**Converted to open gastrectomy**								
No	Ref				Ref			
Yes	1.757 (0.691-4.464)	0.236			1.382 (0.294-6.505)	0.682		
**Transfusion**								
No	Ref				Ref			
Yes	1.393 (0.535-3.628)	0.498			1.382 (0.294-6.505)	0.682		
**Tumor Size (cm)**								
<6	Ref				Ref			
≥6	0.896 (0.500-1.606)	0.731			1.060 (0.389-2.899)	0.909		
**Operation Time (min)**								
<300	Ref		Ref		Ref		Ref	
≥300	2.094 (1.149-3.815)	***0.016***	1.870 (1.004-3.483)	***0.049***	2.557 (0.945-6.918)	***0.065***	2.545 (0.907-7.139)	0.076
**Blood Loss (ml)**								
<200	Ref		Ref		Ref		Ref	
≥200	1.667 (0.931-2.987)	***0.086***	1.484 (0.802-2.747)	0.208	2.771 (0.988-7.773)	***0.053***	2.482 (0.863-7.137)	0.092

ASA, American Society of Anesthesiologists; BMI, Body mass index. Italicized and bold values represent significant differences.

## Discussion

In our study, NACT did not increase the operation time, blood loss, transfusion during or after surgery or the rate of conversion to open surgery. Although NACT could trigger stomach and metastatic lymph node fibrosis (27) and the tissues of patients with NACT are more likely to bleed (28), the laparoscopic monitoring amplification effect, careful intraoperative procedures and the use of laparoscopic high-resolution imaging help reduce unnecessary damage to prevent accidental bleeding. The wide application of intraoperative ultrasound scalpels can also effectively solve these problems. Therefore, no increase in surgical difficulty was noted after chemotherapy.

Lymph node dissection is a key radical gastrectomy for advanced AEG, and the number of lymph nodes dissected is an important prognostic factor for the surgical treatment of advanced gastric cancer (29). In our study, the average number of lymph nodes dissected in both groups of patients undergoing radical resection was greater than 30, which meets the requirements of current guidelines suggesting that LG is feasible in lymph node dissection (30). No significant difference in the number of dissected lymph nodes was noted between the two groups. The number of metastatic LNs was significantly lower in the NACT group (median 0 *vs* 5). After NACT, 5% of the total MLNs could achieve complete tumor regression (31), which may explain the difference.

Postoperative complications are the main indicator for evaluating the safety and feasibility of surgery. In our analysis, the incidence of postoperative complications in the NACT group was slightly higher than that in the LG group; however, the difference was not significant (29.3% *vs.* 25.6%, P= 0.535). Further analysis showed no difference in systemic complications, local complications, minor complications (CD grade<3) or major complications (CD grade≥3). Pulmonary complications obviously accounted for most of the complications in our study, and no difference was noted between the two groups. In their stratified analysis of 92 patients after PSM, Amir et al. (32) found that the NACT group had similar postoperative complications with the surgery alone group. However, in a study of 90 patients, Wei et al. (33) revealed that the NACT group had a higher risk of postoperative infectious complications. Possible explanations for the differences may be that the baselines of the two studies were inconsistent. The cT stage and cN stage in Amir’s study were matched well; however, Wei’s study did not take this factor into consideration. Indeed, a reduction in tumor volume allows less extensive procedures, and nutritional improvement before surgery is helpful to reduce the incidence of complications. Although chemotherapy-induced tissue fibrosis can make surgery more difficult (27) and perhaps increase postoperative complications, LG can provide visual magnification, better exposure, and more detailed organ, blood vessel, and nerve operations, reducing unnecessary intra-operative damage. These problems can be effectively solved by laparoscopy. All patients in this study followed a 3-week rest and nutritional support programme after completing preoperative NACT before surgery. Furthermore, we also performed subgroup analysis to further evaluate complications in different parameters. The results of subgroup analyses showed no significant increase in all types of complications of NACT compared with LG.

Patients with progressive disease and stable disease after neoadjuvant chemotherapy represent a special group of patients, and few studies have been conducted on this group before. However, previous studies (34–37) have shown that approximately 32.1% to 58% of patients inevitably underwent SD or PD after neoadjuvant chemotherapy based on fluorouracil + oxaliplatin, so it is necessary to study the short-term efficacy of this group of patients. Subgroup analysis of complications showed no significant difference in the complications between the SD and PD groups compared with either the PR group or LG group (**Appendix, Tables 1, 2**). Subgroup analysis of postoperative results revealed no significant differences in operative time, intraoperative blood loss or other results between the SD and PD groups compared with either the PR group or LG group (**Appendix, Tables 3, 4**). We also noticed a significant increase in the transfer rate of open abdominal surgery and a longer incisional length in the SD and PD groups compared with the direct LG group (**Appendix, Table 4**). In the SD and PD groups, neoadjuvant chemotherapy was not effective, and some patients experienced tumor progression. Moreover, the oedema of tissues around tumors and metastatic lymph nodes might increase the difficulty of laparoscopic surgery, thus increasing the conversion rate of laparotomy. The increase in the rate of conversion to laparotomy subsequently increased the incision length.

In our analysis, NACT was not an independent risk factor for total complications or for major complications in advanced AEG laparoscopic therapy; thus, the applicability of LG for patients after NACT was further verified. An operation time ≥ 300 minutes was identified as a risk factor for overall complications. A longer operation time always indicates a more complicated situation. In addition, prolonged anaesthesia increases the risk of postoperative complications. According to published studies, old age is a leading risk factor for postoperative complications in gastric cancer surgery (38–40). In our study, old age was an independent risk factor for major complications rather than for overall complications. The reason for this difference may be that LG can effectively reduce the total complications in elderly patients. A previous meta-analysis showed that LG could effectively reduce total complications and minor complications (41). We should also realize that gastrectomy still has higher risks of major complications for elderly patients, and more attention should be given when this procedure is used in elderly patients in clinical practice.

In this study, we also compared the total hospital stay, postoperative hospital stay and time to first flatus between the two procedures. The results showed that the hospital stay was significantly longer in the NACT group. The reason for this finding may be that complications in the NACT group were slightly higher than those in the LG group, and surgeons took a longer time to manage the complications. However, the time to first flatus of the NACT group was significantly shorter than that of the LG group. To investigate whether the difference in the time to first flatus was related to the anastomosis method, we performed a statistical analysis of the anastomosis method between the two groups (**Appendix, Tables 5–7**). We first conducted statistics on the two groups of anastomosis methods. The results revealed no significant difference between the NACT group and the LG group (**Appendix, Table 5**). Then, we compared the time to first flatus of the two most common anastomosis methods within the two groups. The results indicated no significant difference in the time to first flatus of end-to-side anastomosis and semi-end-to-end anastomosis in either the NACT group or the LG group (**Appendix, Table 6**). In addition, in a previous study at our centre, 176 cases of end-to-side esophagojejunostomy and 92 cases of semi-end-to-end esophagojejunostomy were included and compared, and no significant difference in the first time to flatus was noted between the two groups (P = 0.957) (42). Finally, we performed an intergroup comparison between the NACT group and the LG group. The results revealed that the time to first flatus of end-to-side anastomosis in the NACT group was significantly shorter than that in the LG group, and the time to first flatus of semi-end-to-end anastomosis in the NACT group was also significantly shorter than that in the LG group (**Appendix, Table 7**). Therefore, we hypothesized that the difference in the time to first flatus between the NACT group and the LG group was caused by neoadjuvant chemotherapy rather than the difference in anastomosis. In our study, the BMI of the NACT group before surgery was significantly greater than that on admission. AEG is often accompanied by symptoms of obstruction, leading to poor preoperative nutritional status. NACT can effectively improve the obstruction state preoperatively supplemented with enteral nutrition preparation and prove the preoperative nutritional status.

Nevertheless, there are several limitations in the current study. First, as a retrospective analysis conducted at a single centre, this study is subject to possible selection bias despite the use of PSM to reduce bias, which was intended to mimic randomized controlled trials. Second, the regimens and indications for NACT were not standardized; therefore, the effects of different NACT regimens were not analysed.

In conclusion, the findings of this study suggest that NACT combined with LG is safe and feasible in treating locally advanced Siewert type II and III AEG in terms of morbidity and short-term surgical outcomes. Multicentre, prospective, clinical trials with large sample sizes are still warranted to verify our findings.

## Data Availability Statement

The original contributions presented in the study are included in the article/**Supplementary Material**. Further inquiries can be directed to the corresponding author.

## Ethics Statement

The studies involving human participants were reviewed and approved by Ethics Committee of Southwest Hospital (Chongqing, China). Written informed consent for participation was not required for this study in accordance with the national legislation and the institutional requirements.

## Author Contributions

YS and P-wY contributed to the conception and design of the study. M-sD, FQ, Y-Lz, and YW organized the database. DL and QF performed the statistical analysis. QF wrote the first draft of the manuscript. X-sW and Z-sL wrote sections of the manuscript. All authors contributed to the article and approved the submitted version.

## Funding

This study was funded by the Special Science and Technology Innovation Foundation of Social Programs and Livelihood Insurance of Chongqing: Research and application of precise minimally invasive therapy for gastrointestinal malignant tumors (cstc2017shmsA10003-ztzx10001-4).

## Conflict of Interest

The authors declare that the research was conducted in the absence of any commercial or financial relationships that could be construed as a potential conflict of interest.

## Publisher’s Note

All claims expressed in this article are solely those of the authors and do not necessarily represent those of their affiliated organizations, or those of the publisher, the editors and the reviewers. Any product that may be evaluated in this article, or claim that may be made by its manufacturer, is not guaranteed or endorsed by the publisher.
