# Supplementary material for: Short-Term Clinical Efficacy of Neoadjuvant Chemotherapy Combined With Laparoscopic Gastrectomy for Locally Advanced Siewert Type II and III Adenocarcinoma of the Esophagogastric Junction: A Retrospective, Propensity Score-Matched Study
Source: Front Oncol. 2021 Sep 29;11:690662. doi: 10.3389/fonc.2021.690662 (PMC8511681; doi:10.3389/fonc.2021.690662)
Supplement: Supplementary file 1 [file Table_1.docx]

**APPENDIX**

| **Table 1. Postoperative Complications of the patients in the PR group and SD, PD group,n (%).** | | | |
| --- | --- | --- | --- |
| **Variable** | **PR group (n=27)** | **SD, PD group (n=14)** | **P value** |
| **Postoperative complications** | 10(37.0%) | 2(14.3%) | 0.248 |
| **Systemic complication** | 8(29.6%) | 2(14.3%) | 0.483 |
| Heart failure | 1(3.7%) | 0 | 1.000 |
| Respiratory failure | 1(3.7%) | 1(7.1%) | 1.000 |
| Pulmonary infection | 4(14.8%) | 2(14.3%) | 1.000 |
| Pleural effusion | 3(11.1%) | 0 | 0.539 |
| **Local complication** | 5(18.5%) | 0 | 0.146 |
| Duodenal stump leakage | 1(3.7%) | 0 | 1.000 |
| Anastomotic leakage | 2(7.4%) | 0 | 0.539 |
| Intra-abdominal infection | 3(11.1%) | 0 | 0.539 |
| **Infectious complication** | 8(29.6%) | 2(14.3%) | 0.483 |
| **Clavien-Dindo Classification** |  |  |  |
| Grades I-II | 7(25.9%) | 1(7.1%) | 0.306 |
| Grade I | 1(3.7%) | 0 | 1.000 |
| Grade II | 6(22.2%) | 1(7.1%) | 0.389 |
| Grades III-V | 3(11.1%) | 1(7.1%) | 1.000 |
| Grade III | 1(3.7%) | 0 | 1.000 |
| Grade IV | 2(7.4%) | 1(7.1%) | 1.000 |
| Grade V | 0 | 0 | NA |
| **Reoperation** | 0 | 1(7.1%) | 0.341 |
| PR: partial remission; SD: stable disease; PD: progressive disease; NA: not available. | | | |

| **Table 2. Postoperative Complications of the patients in the PR group and SD, PD group,n (%).** | | | |
| --- | --- | --- | --- |
| **Variable** | **PR group (n=27)** | **SD, PD group (n=14)** | **P value** |
| **Postoperative complications** | 10(37.0%) | 2(14.3%) | 0.248 |
| **Systemic complication** | 8(29.6%) | 2(14.3%) | 0.483 |
| Heart failure | 1(3.7%) | 0 | 1.000 |
| Respiratory failure | 1(3.7%) | 1(7.1%) | 1.000 |
| Pulmonary infection | 4(14.8%) | 2(14.3%) | 1.000 |
| Pleural effusion | 3(11.1%) | 0 | 0.539 |
| **Local complication** | 5(18.5%) | 0 | 0.146 |
| Duodenal stump leakage | 1(3.7%) | 0 | 1.000 |
| Anastomotic leakage | 2(7.4%) | 0 | 0.539 |
| Intra-abdominal infection | 3(11.1%) | 0 | 0.539 |
| **Infectious complication** | 8(29.6%) | 2(14.3%) | 0.483 |
| **Clavien-Dindo Classification** |  |  |  |
| Grades I-II | 7(25.9%) | 1(7.1%) | 0.306 |
| Grade I | 1(3.7%) | 0 | 1.000 |
| Grade II | 6(22.2%) | 1(7.1%) | 0.389 |
| Grades III-V | 3(11.1%) | 1(7.1%) | 1.000 |
| Grade III | 1(3.7%) | 0 | 1.000 |
| Grade IV | 2(7.4%) | 1(7.1%) | 1.000 |
| Grade V | 0 | 0 | NA |
| **Reoperation** | 0 | 1(7.1%) | 0.341 |
| PR: partial remission; SD: stable disease; PD: progressive disease; NA: not available. | | | |

| **Table 3. Comparison of operative and postoperative parameters between the PR group and SD, PD group, n (%).** | | | |
| --- | --- | --- | --- |
| **Variable** | **PR group (n=27)** | **SD*, PD group (n=14)** | **P value** |
| **Resection** |  |  | 1.000 |
| R0 | 26(96.3%) | 14(100%) |  |
| R1 | 1(3.7%) | 0 |  |
| **Operation time, min (median ,IQR)** | 289(234,305) | 293(269.25,305) | 0.752 |
| **Blood loss, mL (median ,IQR)** | 160(110,200) | 155(105,370) | 0.879 |
| **Blood transfusion** |  |  | 1.000 |
| Yes | 3(11.1%) | 2(14.3%) |  |
| No | 24(88.9%) | 12(85.7%) |  |
| **Lymph nodes dissection range** |  |  | 0.539 |
| D2 | 25(92.6%) | 14(100%) |  |
| D2+ | 2(7.4%) | 0 |  |
| **Conversion to open from laparoscopic gastrectomy** | 1(3.7%) | 5(35.7%) | 0.013 |
| **Length of incision, cm (median ,IQR)** | 7(6,8) | 7.5(6.75,15.75) | 0.059 |
| **Distal margin, cm (median ,IQR)** | 8(5,12) | 11.5(5,15) | 0.638 |
| **Proximal margin, cm (median ,IQR)** | 3(2,4) | 2.75(2,5) | 0.420 |
| **Tumour size, cm (median ,IQR)** | 3(2,3) | 3.5(2,5.25) | 0.224 |
| **The number of resected lymph nodes (mean ± SD)** | 33.35±12.54 | 34.29±17.75 | 0.228 |
| **The number of metastatic lymph nodes (median ,IQR)** | 0(0,4) | 2(0,7) | 0.085 |
| **Total hospital stay, d (median ,IQR)** | 18(14,23) | 16(12.75,20) | 0.160 |
| **Postoperative hospital stay, d (median ,IQR)** | 13(9,15) | 9.5(8,13.25) | 0.086 |
| **Time to first flatus, d (median ,IQR)** | 3(3,4) | 3.5(3,5) | 0.255 |
| PR: partial remission; SD*: stable disease; PD: progressive disease; SD: standard deviation; IQR: interquartile range. Italicized and bold values represent significant differences. | | | |

| **Table 4. Comparison of operative and postoperative parameters between the LG group and SD, PD group, n (%).** | | | |
| --- | --- | --- | --- |
| **Variable** | **LG group (n=123)** | **SD*, PD group  (n=14)** | **P value** |
| **Resection** |  |  | 1.000 |
| R0 | 115(93.5%) | 14(100%) |  |
| R1 | 8(6.5%) | 0 |  |
| **Operation time, min (median ,IQR)** | 275(243,300) | 293(269.25,305) | 0.275 |
| **Blood loss, mL (median ,IQR)** | 160(110,200) | 155(105,370) | 0.436 |
| **Blood transfusion** |  |  | 1.000 |
| Yes | 13(10.6%) | 2(14.3%) |  |
| No | 110(89.4%) | 12(85.7%) |  |
| **Lymph nodes dissection range** |  |  | 1.000 |
| D2 | 119(96.7%) | 14(100%) |  |
| D2+ | 4(3.3%) | 0 |  |
| **Conversion to open from laparoscopic gastrectomy** | 12(9.8%) | 5(35.7%) | ***0.016*** |
| **Length of incision, cm (median ,IQR)** | 6(5,8) | 7.5(6.75,15.75) | ***0.013*** |
| **Distal margin, cm (median ,IQR)** | 8(5,10) | 11.5(5,15) | 0.194 |
| **Proximal margin, cm (median ,IQR)** | 3(2,3) | 2.75(2,5) | 0.832 |
| **Tumour size, cm (median ,IQR)** | 4(3,5) | 3.5(2,5.25) | 0.171 |
| **The number of resected lymph nodes (mean ± SD)** | 33.68±13.42 | 34.29±17.75 | 0.843 |
| **The number of metastatic lymph nodes (median ,IQR)** | 5(1,9) | 2(0,7) | 0.064 |
| **Total hospital stay, d (median ,IQR)** | 15(13,18) | 16(12.75,20) | 0.469 |
| **Postoperative hospital stay, d (median ,IQR)** | 9(8,11) | 9.5(8,13.25) | 0.595 |
| **Time to first flatus, d (median ,IQR)** | 4(3,5) | 3.5(3,5) | 0.476 |
| LG: laparoscopic gastrectomy; SD*: stable disease; PD: progressive disease; SD: standard deviation; IQR: interquartile range. Italicized and bold values represent significant differences. | | | |

| **Table 5. Comparison of the time of first flatus between the NACT group and LG group, n (%).** | | | |
| --- | --- | --- | --- |
| **Variable** | **NACT group (n=41)** | **LG group (n=123)** | **P value** |
| **Anastomosis methods** |  |  | 0.403 |
| End-to-side esophagojejunostomy | 22(53.7%) | 74(60.2%) |  |
| Semi-end-to-end esophagojejunostomy | 16(39.0%) | 37(30.1%) |  |
| End-to-end esophagojejunostomy | 1(2.4%) | 9(7.3%) |  |
| Side-to-side esophagojejunostomy | 2(4.9%) | 3(2.4%) |  |
| NACT: neoadjuvant chemotherapy; LG: laparoscopic gastrectomy. | | | |

| **Table 6. Comparison of the time to first flatus between the End-to-side esophagojejunostomy and Semi-end-to-end esophagojejunostomy in the NACT group and LG group.** | | | |
| --- | --- | --- | --- |
| **Variable** | **End-to-side esophagojejunostomy** | **Semi-end-to-end esophagojejunostomy** | **P value** |
| **The time to first flatus of the NACT group (median ,IQR)** | 3(3,4) | 3(3,4) | 0.549 |
| **The time to first flatus of the LG group (median ,IQR)** | 4(3,5) | 5(3,5.5) | 0.563 |
| NACT: neoadjuvant chemotherapy; LG: laparoscopic gastrectomy; IQR: interquartile range. | | | |

| **Table 7. Comparison of the time to first flatus between the NACT group and LG group.** | | | |
| --- | --- | --- | --- |
| **Variable** | **NACT group** | **LG group** | **P value** |
| **The time to first flatus of End-to-side esophagojejunostomy (median ,IQR)** | 3(3,4) | 4(3,5) | ***0.039*** |
| **The time to first flatus of Semi-end-to-end**  **esophagojejunostomy (median ,IQR)** | 3(3,4) | 5(3,5.5) | ***0.022*** |
| NACT: neoadjuvant chemotherapy; LG: laparoscopic gastrectomy; IQR: interquartile range. Italicized and bold values represent significant differences. | | | |
